# Factors associated with severe maternal morbidity in Kelantan, Malaysia: A comparative cross-sectional study

**DOI:** 10.1186/s12884-016-0980-2

**Published:** 2016-07-26

**Authors:** Mohd Noor Norhayati, Nik Hussain Nik Hazlina, Abd. Aziz Aniza, Zaharah Sulaiman

**Affiliations:** 1 Department of Family Medicine, School of Medical Sciences, Universiti Sains Malaysia, Health Campus, Kubang Kerian, Kelantan 16150 Malaysia; 2 Women’s Health Development Unit, School of Medical Sciences, Universiti Sains Malaysia, Health Campus, Kubang Kerian, Kelantan 16150 Malaysia; 3 Faculty of Medicine, Universiti Sultan Zainal Abidin, Medical Campus, Jalan Sultan Mahmud, Kuala Terengganu, Terengganu 20400 Malaysia

**Keywords:** Severe maternal morbidity, Pregnancy complications, Associated factors, Comparative studies

## Abstract

**Background:**

Knowledge on the factors associated with severe maternal morbidity enables a better understanding of the problem and serves as a foundation for the development of an effective preventive strategy. However, various definitions of severe maternal morbidity have been applied, leading to inconsistencies between studies. The objective of this study was to identify the sociodemographic characteristics, medical and gynaecological history, past and present obstetric performance and the provision of health care services as associated factors for severe maternal morbidity in Kelantan, Malaysia.

**Methods:**

A comparative cross-sectional study was conducted in two tertiary referral hospitals in 2014. Postpartum women with severe morbidity and without severe morbidity who fulfilled the inclusion and exclusion criteria were eligible as cases and controls, respectively. The study population included all postpartum women regardless of their age. Pregnancy at less than 22 weeks of gestation, more than 42 days after the termination of pregnancy and non-Malaysian citizens were excluded. Consecutive sampling was applied for the selection of cases and for each case identified, one unmatched control from the same hospital was selected using computer-based simple random sampling. Simple and multiple logistic regressions were performed using Stata Intercooled version 11.0.

**Results:**

A total of 23,422 pregnant women were admitted to these hospitals in 2014 and 395 women with severe maternal morbidity were identified, of which 353 were eligible as cases. An age of 35 or more years old [Adj. *OR* (95 % CI): 2.6 (1.67, 4.07)], women with past pregnancy complications [Adj. *OR* (95 % CI): 1.7 (1.00, 2.79)], underwent caesarean section deliveries [Adj. *OR* (95 % CI): 6.8 (4.68, 10.01)], preterm delivery [Adj. *OR* (95 % CI): 3.4 (1.87, 6.32)] and referral to tertiary centres [Adj. *OR* (95 % CI): 2.7 (1.87, 3.97)] were significant associated factors for severe maternal morbidity.

**Conclusions:**

Our study suggests the enhanced screening and monitoring of women of advanced maternal age, women with past pregnancy complications, those who underwent caesarean section deliveries, those who delivered preterm and the mothers referred to tertiary centres as they are at increased risk of severe maternal morbidity. Identifying these factors may contribute to specific and targeted strategies aimed at tackling the issues related to maternal morbidity.

## Background

In Malaysia, pregnant women are entitled to a wide range of medical services that include antenatal care, hospital delivery, caesarean sections, and the medical and surgical treatment of complications. Low-risk pregnant mothers are allowed to deliver in alternative birthing centres adjacent to primary health clinics, secondary or tertiary health centres. High-risk pregnant mothers are seen by family medicine specialists or referred to tertiary centres. Improvements in the health care delivery system, the implementation of risk approach strategies and the confidential enquiry into maternal deaths have been used for many years in Malaysia [1]. As a result, the maternal mortality ratio has been reduced, but it has plateaued for more than 20 years [1] and no further reduction has been seen which indicates the need for a new approach.

The literature on severe maternal morbidity has revealed several contributing factors for severe maternal morbidity. Employment status [2], low household income [3], a previous history of abortion [3], multiple births [4] and minimal antenatal care [3] were documented contributing factors for severe maternal morbidity. Mixed findings were reported in relation to age [5, 6], race [4, 7], educational level [3, 7], co-existing medical conditions [8, 9], parity [9, 10], period of gestation [5, 9], mode of delivery [9, 11], previous caesarean section [8, 12] and pre-pregnancy body mass index [4, 8]. It is important to note that the studies applied a variety of definitions for severe maternal morbidity and the consolidation of information among the studies was, therefore, difficult.

Severe maternal morbidity refers to ‘potentially life-threatening conditions during pregnancy, childbirth or after termination of pregnancy from which maternal near miss cases would emerge’ [13, 14]. The World Health Organization (WHO) criteria not only consider the clinical disorders (haemorrhagic, hypertensive and other systemic disorders) but also the severe management indicators to demonstrate the severity and to enhance the identification of severe maternal morbidity [13].

Ascertaining associated factors for severe maternal morbidity enables a better understanding of the problem and serves as a foundation for the development of an effective preventive strategy. This refers to primary prevention through screening or prevention at the institutional, provider and client levels that may ultimately prevent a condition from causing death or severe morbidity [15]. The aim of this study was to identify the sociodemographic characteristics, medical and gynaecological history, past and present obstetric performance and the provision of health care services as factors associated with severe maternal morbidity in Kelantan, Malaysia. We hypothesized that these factors were significantly related to the occurrence of severe maternal morbidity.

## Methods

In a comparative cross-sectional study conducted in Raja Perempuan Zainab II Hospital and Universiti Sains Malaysia Hospital, data from postpartum women in the year 2014 were obtained. These hospitals are the two referral and tertiary hospitals in Kelantan, Malaysia. Postpartum women with severe morbidity and without severe morbidity who fulfilled the inclusion and exclusion criteria were eligible as cases and controls, respectively. The study population included all postpartum women regardless of age. Pregnancy at less than 22 weeks of gestation, more than 42 days after termination of pregnancy and non-Malaysian citizens were excluded.

Sample size was estimated by comparing two proportions for categorical variables and comparing two means for numerical variables using the Power and Sample Size calculation software version 3.0.43 (Microsoft Corp., 2012). The variable that yielded the biggest sample size for this objective was that of co-existing medical conditions. The proportion of co-existing medical conditions among women without severe morbidity was 0.08 [4]. A detectable odds ratio of 2 was decided after considering its clinical importance and the minimum required sample size was 340. After considering the non-response rate of 10 %, the sample size estimated for each group of cases and controls was 374. Consecutive sampling was applied for the selection of cases until the sample size was met. For each case identified, one unmatched control from the same hospital was selected using computer-based simple random sampling [16] from the predefined estimate of daily deliveries. The sampling method of controls overcame the non-probability sampling limitations in this study.

A total of 23,422 pregnant women were admitted to these hospitals in 2014. A nursing-trained research assistant prospectively reviewed the admission registers and medical records in delivery rooms and obstetrics and gynaecology wards daily. Information on the sociodemographic characteristics, current obstetric history, clinical parameters, past obstetric history, medical and gynaecological history, foetal outcome and the provision of health care were obtained from hospital and home-based medical records during hospitalization.

The data were entered using IBM SPSS Statistics version 22.0 (SPSS Inc., 2013) and analysed using Stata Intercooled version 11.0 (Stata Corp., 2003). The data were checked and filtered before analysis. Simple and multiple logistic regressions were used to identify the associated factors for severe maternal morbidity. The dependent variable was maternal morbidity status that categorized women with and without severe maternal morbidity. The independent variables included in the analyses were selected a priori based on the literature and clinical knowledge that supported these as potential risk factors for severe maternal morbidity. The following variables were assessed: (i) sociodemographic factor (age, race, marital status, education level and occupation), (ii) medical and gynaecological history (comorbidities and history of abortion), (iii) past and present obstetric history [parity, past pregnancy complications, multiple births, period of gestation, antenatal care booking, antenatal care visits, mode of delivery, history of caesarean section deliveries and body mass index (BMI) at booking] and (iv) the provision of health care services (referral from health centres).

The categorization of variables was performed according to clinical application of the selected variables. Age was categorized into younger (<35 years) and older (≥35 years) maternal age [17]. The period of gestation in the present study was categorized into term (≥37 weeks) and preterm (<37 weeks) [18]. Antenatal care booking was categorized into early (≤12 weeks) and late (>12 weeks). The number of antenatal visits was categorized in accordance with the recommendations of the Malaysian Ministry of Health. A minimum of eight visits throughout the pregnancy was considered as optimum and seven or less as suboptimum [19], while BMI at booking was categorized into normal (18.50–24.99 kg/m^2^), underweight (≤18.49 kg/m^2^), overweight (25.00–29.99 kg/m^2^) and obese (≥30.00 kg/m^2^) [20]. Comorbidity was considered to be present when there were pre-existing medical conditions reported such as hypertension, diabetes, asthma, heart diseases, thyroid disorders and psychiatric disorders.

Backward stepwise and manual backward procedures were performed for variable selection. This process of deleting, refitting and verifying continued until it appeared that all the important variables were included in the model and those variables excluded were clinically and/or statistically unimportant. The continuous variables were checked for linearity in logit. Interaction terms were created and tested in the model for improved model fit. Multicollinearity was assessed through analysis of the correlation coefficients in the correlation matrix, standard errors of parameters and confidence interval of estimated regression coefficients.

The model fit was assessed by plotting the predicted probabilities using the receiver operating characteristics (ROC), Pearson chi square and Hosmer and Lemeshow goodness of fit tests. Diagnostic assessment to identify potential influential covariate patterns were performed using Delta chi-square (∆χ^2^), Delta deviance (∆D) and Pregibon delta-beta (∆β).The potential cases from specific covariate pattern identified as influential in influential statistics were tested manually by removing and checking for beta coefficient change. Interpretations were based on proportional odds model. Findings were presented with crude and adjusted odds ratio (*OR*), 95 % confidence interval (CI) and *P* value. Level of significance was set at 0.05 with two tailed fashion.

## Results

There were a total of 21,756 deliveries, 21,579 live births and 395 women with severe maternal morbidity in 2014. However, 42 cases were excluded as they were at less than 22 weeks of gestation (*n* = 32) and were non-Malaysian citizens (*n* = 10). A total of 353 cases were eligible and accordingly, 353 women without severe maternal morbidity were identified as controls. However, one case (A339) and one control (B283) were unbooked (no antenatal check-up), therefore, they were not included in the analyses due to high missing data related to gestation, booking and antenatal care visits. The final response rate was 99.7 % (352/353) for both groups and overall population studied.

The number of respondents that were suitable for analysis (*n* = 352 per group) was lower than the calculated sample size (*n* = 374 per group). Therefore, the post-hoc power of the study with 352 participants per group was recalculated using the Power and Sample Size Calculation software version 3.0.43 (Microsoft Corp., 2012) for comparing two proportions. The recalculated power was 81.3 %, which was acceptable.

### Characteristics of participants

Compared with women without severe maternal morbidity, women with severe maternal morbidity were more often older than 35 years, had tertiary-level education and had undergone a previous caesarean section (Table 1 and Table 2). While only 25.3 % of women without severe maternal morbidity required caesarean section, 78.1 % of women with severe maternal morbidity required one. Women with severe maternal morbidity had a significantly longer duration of hospitalization with a mean (SD) of 5.8 (4.30) days compared with 3.0 (1.74) days for women without severe maternal morbidity (*P* <0.001).

**Table 1:** Sociodemographic, medical and gynaecological history and provision of health care services profiles of women with (cases) and without (controls) severe maternal morbidity

Variables	Severe maternal morbidity (*n* = 352)		Non-severe maternal morbidity (*n* = 352)	
	*n*	(%)	*n*	(%)
*Sociodemographic*				
Age				
<35 years	235	(66.8)	296	(84.1)
≥35 years	117	(33.2)	56	(15.9)
Race				
Malays	349	(99.1)	350	(99.4)
Others	3	(0.9)	2	(0.6)
Marital status				
Married	349	(99.1)	352	(100.0)
Single	3	(0.9)	0	(0)
Education level				
Nil and Primary	15	(4.3)	13	(3.7)
Secondary	216	(61.4)	241	(68.5)
Tertiary	121	(34.4)	98	(27.8)
Occupation				
Unemployed	186	(52.8)	199	(56.5)
Self-employed	25	(7.1)	32	(9.1)
Support group	89	(25.3)	90	(25.6)
Professional	52	(14.8)	31	(8.8)
Husband education				
Primary	11	(3.2)	5	(1.4)
Secondary	229	(65.6)	254	(72.2)
Tertiary	109	(31.2)	93	(26.4)
Husband occupation				
Unemployed	6	(1.7)	5	(1.4)
Self-employed	132	(37.8)	158	(44.9)
Support group	168	(48.1)	167	(47.4)
Professional	43	(12.3)	22	(6.3)
*Medical and gynaecological history*				
Comorbidity				
Absent	308	(87.5)	326	(92.6)
Present	44	(12.5)	26	(7.4)
h/o abortion				
Absent	277	(78.7)	274	(77.8)
Present	75	(21.3)	78	(22.2)
*Provision of health care services*				
Health care facility				
Raja Perempuan Zainab II Hospital	274	(77.8)	274	(77.8)
Universiti Sains Malaysia Hospital	78	(22.2)	78	(22.2)
Referral status				
Not referred	102	(29.0)	240	(68.2)
Referred	250	(71.0)	112	(31.8)
Birth attendant				
Doctors	335	(95.2)	197	(56.0)
Midwifes	17	(4.8)	155	(44.0)

**Table 2:** Past and present obstetric history of women with (cases) and without (controls) severe maternal morbidity

Variables	Severe maternal morbidity (*n* = 352)				Non-severe maternal morbidity (*n* = 352)			
	mean	(SD^a^)	*n*	(%)	mean	(SD^a^)	*n*	(%)
*Past and present obstetric history*								
Number of children	2.1	(2.33)			1.7	(1.80)		
Parity	3.0	(2.29)			2.7	(1.81)		
Gestational age at booking (week)			13.1	(5.65)			13.0	(4.98)
Booking								
Early (≤12 weeks)			190	(54.0)			186	(52.8)
Late (>12 weeks)			162	(46.0)			166	(47.2)
Birth spacing (year)^b^			4.6	(3.04)			3.8	(2.27)
BMI at booking (kg/m^2^)								
Normal			116	(33.0)			157	(44.6)
Underweight			20	(5.7)			47	(13.4)
Overweight			105	(29.8)			90	(25.6)
Obese			111	(31.5)			58	(16.5)
Antenatal care visits								
Optimum (≥8 visits)			313	(88.9)			334	(94.9)
Suboptimum (<7 visits)			39	(11.1)			18	(5.1)
h/o caesarean section								
Absent			266	(75.6)			305	(86.6)
Present			86	(24.4)			47	(13.4)
Past pregnancy complications								
Absent			269	(76.4)			314	(89.2)
Present			83	(23.6)			38	(10.8)
Period of gestation								
Term (≥37 weeks)			253	(71.9)			334	(94.9)
Preterm (<37 weeks)			99	(28.1)			18	(5.1)
Mode of delivery								
vaginal			77	(21.9)			263	(74.7)
caesarean section			275	(78.1)			89	(25.3)
Colour code								
White			18	(5.1)			51	(14.5)
Green			255	(72.4)			261	(74.1)
Yellow			65	(18.5)			37	(10.5)
Red			14	(4.0)			3	(0.9)
Fetal sex								
Boy			178	(50.6)			190	(54.0)
Girl			173	(49.1)			162	(46.0)
Ambigous			1	(0.3)			0	(0.0)
Fetal viability								
Alive			340	(96.6)			350	(99.4)
Dead			12	(3.4)			2	(0.6)
Multiple births								
No			343	(97.4)			352	(100.0)
Yes			9	(2.6)			0	(0.0)

*Note.* BMI = body mass index

^a^Standard deviation

^b^Available for 233 cases and 238 controls

The numbers of samples in the groups for race, marital status and multiple births were small. In addition, there were high missing data for birth spacing in women with no records of a previous pregnancy. These four variables were less clinically important than those identified in the previous literature. Therefore, these variables were not included in the logistic regression analyses. The overall BMI ranges from 13.1 to 59.7 kg/m^2^. In controls, it ranges from 14.2 to 47.5 kg/m^2^ and in cases, it ranges from 13.1 to 59.7 kg/m^2^.

### Simple logistic regression analysis

In total, 14 variables were chosen for descriptive analysis based on their clinical importance and completeness of data (Table 3). Simple logistic regression screened and identified 12 variables with *P* < 0.3 and two highly insignificant variables i.e. booking and history of abortion. Therefore, 12 variables were included in the variable selection procedures of multiple logistic regression analysis.

**Table 3:** Associated factors for severe maternal morbidity using simple logistic regression

Variable	Crude *OR* ^a^	(95 % *CI* ^b^)	*Wald* stat^c^	*P* value
Parity	1.1	(1.02, 1.18)	5.91	0.015
Age				
<35 years	1.0		29.00	<0.001
≥35 years	2.6	(1.83, 3.78)		
Education level				
Nil and Primary	1.0		3.93	0.141
Secondary	0.8	(0.36, 1.67)		
Tertiary	1.1	(0.49, 2.36)		
Occupation				
Unemployed	1.0		6.68	0.083
Self-employed	0.8	(0.48, 1.47)		
Support group	1.1	(0.74, 1.51)		
Professional	1.8	(1.10, 2.92)		
Comorbidity				
Absent	1.0		5.19	0.023
Present	1.8	(1.08, 2.98)		
h/o abortion				
Absent	1.0		0.08	0.784
Present	1.0	(0.66, 1.36)		
h/o caesarean section				
Absent	1.0	(1.42, 3.10)	14.27	<0.001
Present	2.1			
Past pregnancy complications				
Absent	1.0		20.62	<0.001
Present	2.5	(1.68, 3.87)		
Booking				
Early (≤12 weeks)	1.0		0.09	0.763
Late (>12 weeks)	0.96	(0.71, 1.28)		
BMI at booking (kg/m^2^)				
Normal	1.0		35.44	<0.001
Underweight	0.6	(0.32, 1.02)		
Overweight	1.6	(1.09, 2.29)		
Obese	2.6	(1.74, 3.86)		
Antenatal care visits				
Optimum (≥8 visits)	1.0		8.60	0.003
Suboptimum (<7 visits)	2.3	(1.30, 4.13)		
Mode of delivery				
Vaginal delivery	1.0		207.24	<0.001
Caesarean section	10.6	(7.44, 14.96)		
Period of gestation				
Term (≥37 weeks)	1.0			<0.001
Preterm (<37 weeks)	7.3	(4.28, 12.31)	72.95	
Referral status				
Not referred	1.0		111.27	<0.001
Referred	5.3	(3.81, 7.24)		

*Note.* BMI = body mass index

^a^Crude odds ratio

^b^Confidence interval

^c^Wald statistic

### Multiple logistic regression analysis

#### Variable selection

There were 12 potential variables included in the multiple logistic regression analysis (Table 4). The stepwise procedure based on Wald statistics produced five significant variables i.e. age, mode of delivery, period of gestation, pregnancy complications and referral status. The significant variables were confirmed by a manual backward procedure based on the log-likelihood ratio (*LR*) test.

**Table 4:** Preliminary main effect model based on multiple logistic regression

Variable	Adjusted *OR* ^a^	(95 % *CI* ^b^)	*LR* stat^c^	*P* value
Parity	1.0	(0.87, 1.11)	0.11	0.744
Age				
<35 years	1.0		18.36	<0.001
≥35 years	2.6	(1.67, 4.07)		
Education level				
Nil and Primary	1.0		5.06	0.080
Secondary	0.9	(0.33, 2.68)		
Tertiary	1.7	(0.54, 5.08)		
Occupation				
Unemployed	1.0		3.49	0.322
Self-employed	0.8	(0.36, 1.59)		
Support group	0.9	(0.56, 1.39)		
Professional	1.5	(0.83, 2.83)		
Comorbidity				
Absent	1.0		1.01	0.316
Present	1.4	(0.72, 2.71)		
h/o caesarean section				
Absent	1.0		2.40	0.121
Present	0.6	(0.35, 1.13)		
Past pregnancy complications				
Absent	1.0		3.92	0.048
Present	1.7	(1.00, 2.79)		
BMI at booking (kg/m^2^)				
Normal	1.0		5.22	0.156
Underweight	0.7	(0.35, 1.42)		
Overweight	1.2	(0.76, 1.95)		
Obese	1.6	(0.94, 2.60)		
Antenatal care visits				
Optimum (≥8 visits)	1.0		0.89	0.345
Suboptimum (<7 visits)	1.4	(0.68, 3.03)		
Mode of delivery				
Vaginal delivery	1.0		106.07	<0.001
Caesarean section	6.8	(4.68, 10.01)		
Period of gestation				
Term (≥37 weeks)	1.0		17.82	<0.001
Preterm (<37 weeks)	3.4	(1.87, 6.32)		
Referral status				
Not referred	1.0		27.06	<0.001
Referred	2.7	(1.87, 3.97)		

*Note.* BMI = body mass index

^a^Adjusted odds ratio

^b^Confidence interval

^c^Log - likelihood ratio statistic

#### Checking linearity of the continuous variables

None of the significant variables were numerical variables. Therefore, the linearity of continuous variables by fracpoly, lintrend and design variable methods was not checked.

#### Checking interaction

There were ten possible and clinically important interaction terms tested. There was no significant interaction between the variables (*P* > 0.05).

#### Checking multicollinearity

Multicollinearity among independent variables was assessed using the correlation matrix, standard error and confidence interval. There was a possible multicollinearity suggested by the correlation matrix (*r*) > 0.3 between the mode of delivery and referral status (0.38); however, it was not clinically supported. Moreover, standard error for each associated factor was subjectively small and lesser than their respective beta coefficients. Confidence intervals of the estimated regression coefficient were also narrow. Thus, it can be concluded that multicollinearity did not exist in the model.

#### Checking overall model fitness

High overall correctly classified percentage of 76.4 %, area under the ROC of 84.2 %, non-significance of Pearson chi square (*P* = 0.622) and non-significance of Hosmer and Lemeshow (*P* = 0.840) showed that the model was fit.

#### Checking model diagnostics

The plots of Delta chi-square influential statistics, Delta deviance influential statistics and Pregibon delta-beta statistics versus estimated probability showed a list of covariate patterns as outliers. Delta chi-square versus estimated probability showed that covariate pattern 5 as possible influential outlier. Delta deviance versus estimated probability showed that the covariate pattern 5 as possible influential outlier. Pregibon delta-beta versus estimated probability showed that the covariate pattern 3 and 10 as possible influential outliers.

#### Remedial measures

The potential influential outliers were tested by removing them one by one and checking for the percentage change in the regression coefficient. Only one of five variables in each of the covariate patterns had a maximal change of > 20 %, therefore, it is acceptable to retain the covariate patterns in the model.

#### Final model and interpretation

The final model for associated factors of severe maternal morbidity was the best fit, parsimonious and biologically plausible. Age, past pregnancy complications, mode of delivery, period of gestation and referral status were the significant adjusted variables that influenced the occurrence of severe maternal morbidity (Table 5).

**Table 5:** Associated factors for severe maternal morbidity using multiple logistic regression

Variables	Adjusted *OR* ^a^	(95 % *CI* ^b^)	*LR* stat^c^	*P* value
Age				
<35 years	1.0		18.36	<0.001
≥35 years	2.6	(1.67, 4.07)		
Past pregnancy complications				
Absent	1.0		3.92	0.048
Present	1.7	(1.00, 2.79)		
Mode of delivery				
Vaginal delivery	1.0		106.07	<0.001
Caesarean section	6.8	(4.68, 10.01)		
Period of gestation				
Term (≥37 weeks)	1.0		17.82	<0.001
Preterm (<37 weeks)	3.4	(1.87, 6.32)		
Referral status				
Not referred	1.0		27.06	<0.001
Referred	2.7	(1.87, 3.97)		

^a^Adjusted odds ratio

^b^Confidence interval

^c^Log - likelihood ratio statistic

*Note.* No significant interaction; no multicollinearity problem; model assumptions met; no influential outliers)

## Discussion

The findings of our study will contribute to future comparisons as more studies with similar definitions are published. Previous studies differed from the current study in the definition and criteria applied for severe maternal morbidity. The former variables may behave differently that contributed to limited data for present comparisons using updated definitions. Moreover, cases of severe maternal morbidities are associated with the acute complications and are likely to have characteristics similar to maternal death that warrant better management of care. Therefore, the determination of specific factors involved in severe maternal morbidity cases can provide evidence to further reduce maternal death.

In the present study, the occurrence of severe maternal morbidity was significantly associated with women 35 years old or older, women with past pregnancy complications, those who underwent caesarean section deliveries, those who delivered preterm and cases referred to tertiary centres. These variables, though not amenable, are useful for the identification of women that require extra vigilance in assessing maternal risks.

Pregnancy at an advanced maternal age, defined as 35 years or older, was identified to be strongly associated with severe maternal morbidity. This finding is in agreement with the results of univariable results analysis in the literature [17, 21–24]. Correspondingly, the multivariable analysis in our study showed that the odds of severe maternal morbidity was highly significant with almost three times higher in women with advanced maternal age compared to those of a younger age. In contrast, a study in the intensive care unit in Brazil showed no association between age and severe maternal morbidity [25]. This is because only 7 % of the sample was over 35 years; thus, it was underpowered to evaluate this group of women.

Advanced maternal age was often associated with increased risk of abruption placenta or abnormally invasive placenta [23] along with chronic diseases that gives them a lack of physiological reserve to respond to pregnancy pathology [17, 26, 27] or due to unplanned pregnancy [26, 28]. This could be addressed through better health education and access to contraceptive services. Pregnancies at an advanced maternal age may become a large and growing population due to delayed marriage and career development, which must be tackled through suitable social policies [28]. Adverse maternal risk was also reported at the opposite age extreme of 15 to 19 years old [7]. However, it was not separately analysed in our study due to the very small sample size.

The findings in our study are in accordance with recent literature suggesting that the mode of delivery with particular reference to caesarean section, is an associated factor for severe maternal morbidity [25, 29]. Therefore, it is unsurprising that the high proportion (78.1 %) of women with severe maternal morbidity in our study delivered via caesarean section, as it may be the most effective way of managing a high-risk pregnancy.

In contrast, caesarean section deliveries also carry a higher risk of maternal complications such as haemorrhage, thromboembolism or infection. Previous studies showed that the risk of severe maternal morbidity was around two to five times higher in caesarean section compared to vaginal deliveries [17, 25, 30, 31]. Our results demonstrate that women who delivered via caesarean section had an almost seven times higher odds of experiencing severe maternal morbidity compared to those who delivered vaginally. It is important to note that the caesarean sections in our facilities are performed only when medically indicated.

With regard to the level of health facility, it is also interesting to consider that one cross-sectional study in Tanzania demonstrated a three times higher risk of caesarean section complications in a regional hospital compared to a referral teaching hospital. This finding, however, was observed among maternal near miss cases [32]. Unfortunately, with the current exploratory study design, it remains unclear whether the morbidity related to caesarean section was due to pre-existing conditions that led to the decision to undergo caesarean section or as a consequence from the procedure itself.

Information on women’s previous obstetric history is valuable. Although the studies were conducted with different case definitions, cases of severe maternal morbidity are consistently reported to be more likely to have past obstetric complications [4, 33]. Our results observed that the occurrence of past obstetric complications was doubled in severe maternal morbidity cases than in non-severe maternal morbidity cases. Most of the complications that occurred were related to hypertensive and haemorrhagic disorders and gestational diabetes. Our results also suggest that the odds of severe maternal morbidity were two-fold higher in women with a past history of obstetric complications than those without any past history. However, a more concerning issue is that the past events do not affect family planning decision making of the women leading to the recurrence of severe morbid conditions [34]. A recent study showed that after excluding cases with tubal ligation and hysterectomy among women with severe morbid conditions in the past pregnancies, there was no difference in the proportion of becoming pregnant again within five years between severe morbid women and controls (7.5 % vs 9.3 %) [35].

The findings of our study observed a six-fold higher occurrence of preterm deliveries in women with severe maternal morbidity compared to those without severe maternal morbidity. The preterm deliveries were of a gestational age ranging between 25 to 36 weeks, with most occurring at 34 weeks. Severe preeclampsia, eclampsia and abruptio placenta were major underlying conditions. We also observed an over three-fold increased odds of preterm deliveries than term deliveries in women with severe maternal morbidity. It is plausible that provider-initiated preterm birth might be a consequence of morbidity or to prevent further morbidity, and therefore, it may be a life-saving measure for both mother and foetus. However, the association found between the period of gestation and severe maternal morbidity has not been previously investigated using the same WHO definitions and criteria for diagnosis, thus limiting comparisons.

A recent WHO multicountry survey observed that approximately 76 % of preterm deliveries follow the spontaneous onset of labour and 24 % are provider-initiated. Common maternal conditions such as low maternal height (<145 cm), diabetes and pre-eclampsia contributed to the risk of spontaneous and provider-initiated preterm birth. Only the mode of delivery differed, in which vaginal deliveries were more common in spontaneous preterm birth and caesarean section deliveries were more common in provider-initiated preterm birth [36].

With respect to the provision of health care services, the findings from our study suggested a strong association between referral for delivery and severe maternal morbidity. More than two-thirds (71.0 %) of women with severe maternal morbidity were referred cases implying the severity and deterioration of maternal health. This result is substantially reassuring as the women were high-risk and required referral to higher-level centres. Conversely, the referral to tertiary centres in our study represents an almost three-fold increased odds for severe maternal morbidity compared to women who were not referred. A study conducted in Nigeria using different classification criteria found a four-fold increase of morbid conditions in referred cases due to patients’ delay in seeking care to primary centres in whom the severe complications were already imminent [37].

Referral was described as a complex variable as it also incorporates the health seeking behaviour, the perception of risk by both the women and health care provider and geographical accessibility [38]. Nonetheless, the association found between referral and severe maternal morbidity was not evident in previous studies. Likewise, our results supported the importance of referral to tertiary centres; however, it is beyond the scope our study to quantify the delays in the referral of obstetric cases from peripheral health facilities.

### Strengths and limitations

To the best of our knowledge, this is the first study conducted in Malaysia based on the WHO criteria of severe maternal morbidity that allowed for standard international comparison. Identifying the factors associated with severe maternal morbidity may contribute to improving the current knowledge and to upgrading the existing strategies aimed at tackling the issues related to maternal morbidity. Our study was conducted over an uninterrupted one-year period and the prospective data collection allowed for the clarification of doubts about the record from the health care providers, thus producing more stable estimates of the outcome.

This study has several potential limitations. This study was restricted to two referral tertiary hospitals and does not represent all cases of severe maternal morbidity in Kelantan. Because of the cross-sectional nature of this study, we were unable to make any definitive statement on the direction of causality.

### Recommendation

Surveillance for severe maternal morbidity at the facility or state level could be implemented and the findings could be interpreted in tandem with the review of maternal mortality. The exploration of associated factors for severe maternal morbidity was not meant to determine causality. For example, although there was an association between severe maternal morbidity and mode of delivery, the temporal sequence of events cannot be determined based on the present cross-sectional study design. Future studies with a confirmatory research approach and appropriate design need to be undertaken to establish the causal relationship. This is in the light of the rapidly rising trend of caesarean sections worldwide and the current practice of caesarean sections constituted 20.7 % of deliveries in these two facilities. Limited local data are available for comparison.

## Conclusion

Our study supports the enhanced screening and monitoring of mothers with higher age group, those with past pregnancy complications, those who underwent caesarean section deliveries, those who delivered preterm and cases referred to tertiary centres as they are at increased risk of severe maternal morbidity.

## Abbreviations

BMI, body mass index; CI, confidence interval; *LR*, log-likelihood ratio; *OR*, odds ratio; ROC, receiver operating characteristics; WHO, World Health Organization
